# Association of Male Partners’ Gender-Equitable Attitudes and Behaviors with Young Mothers’ Postpartum Family Planning and Maternal Health Outcomes in Kinshasa, DRC

**DOI:** 10.3390/ijerph191912182

**Published:** 2022-09-26

**Authors:** Anastasia J. Gage, Francine E. Wood, Darling Kittoe, Preethi Murthy, Rianne Gay

**Affiliations:** 1 School of Public Health and Tropical Medicine, Tulane University, New Orleans, LA 70112, USA; 2 Center on Gender Equity and Health, University of California San Diego, La Jolla, CA 92093, USA; 3 Tulane International LLC, Ngaliema, Kinshasa, Democratic Republic of the Congo

**Keywords:** male involvement, maternal health, postpartum family planning, exclusive breastfeeding, gender

## Abstract

Inequitable gender norms can contribute to rapid repeat pregnancies and adverse maternal health outcomes among adolescent girls and young women. This study examined associations between male partners’ gender-equitable attitudes and behaviors and postpartum family planning (FP) and maternal and newborn health (MNH) outcomes among first-time mothers aged 15–24 in Kinshasa, Democratic Republic of the Congo. Participants were 1335 couples who were successfully interviewed in the Momentum project’s 2018 baseline and 2020 endline surveys. Multivariable regression models were used to analyze predictors of postpartum FP discussion and use, shared MNH decision making, completion of the maternal health continuum of care, and exclusive breastfeeding. Male involvement in maternal health was significantly associated with FP discussion and shared decision making. Male partners’ willingness to be involved in routine childcare and shared decision making were significant positive predictors of exclusive breastfeeding. Postpartum FP outcomes were shaped by the intersection of marital status and male partners’ gender-equitable attitudes, intimate partner violence perpetration, and willingness to engage in routine childcare activities to constitute advantage for some outcomes and disadvantage for others. Interventions must use multiple measures to better understand how young mothers’ health outcomes are shaped by their male partners’ gender-related attitudes and behaviors.

## 1. Introduction

It is widely recognized that gender inequality, unequal power dynamics and restrictive gender norms undermine the health of women and girls, men and boys, and gender minorities. Gender refers to “the culturally defined roles, responsibilities, attributes, and entitlements associated with being male or female in a given setting, along with the power relations between and among women and men, and boys and girls” [1] (p. 2375). Evidence indicates that gender inequalities and power imbalances are pervasive, embedded in all facets of society, and greatly affect interpersonal relationships and individual agency [2,3]. As most gender systems are deeply patriarchal and assign higher value to that which is male or masculine than to that which is female or feminine [4,5] and as they reinforce systemic inequality which undermines individuals’ rights and restricts their opportunities, health outcomes for women and girls are disproportionately disadvantaged [6].

Various international conferences and United Nations (UN) instruments have recognized the importance of achieving gender equality and empowering women and girls. The 1993 World Conference on Human Rights recognized violence against women as a human rights violation and public health issue [7]. The 1994 International Conference on Population and Development highlighted women’s empowerment and reproductive rights and the importance of examining gender and gender inequities in sexual and reproductive health [8]. Since then, there has been an increase in programs that reach men and boys alongside women and girls, recognizing that programs involving men lead to improved gender-equitable attitudes among male partners and, consequently, improved family health [9]. The Fourth World Conference on Women achieved global consensus on the Beijing Platform of Action for the advancement of women and the achievement of equality between women and men as a matter of human rights [10]. More recently, the 2030 Agenda for Sustainable Development and Goal 5 of the 17 Sustainable Development Goals (SDG5) recognize that achieving gender equality and empowering all women and girls in all aspects of their lives is integral to a better and more sustainable future for all.

A 2019 *Lancet* series on gender equality, norms, and health contended that the 2030 Agenda for Sustainable Development and the universal health coverage goals will not be achieved unless greater attention is paid to both gender inequalities and the restrictive gender norms that underlie and maintain them [1]. Although significant advances have occurred in family planning (FP) and modern contraceptive uptake over the last two decades, there are still powerful barriers that need to be addressed to meet the demand for effective and safe FP use. As of 2019, 270 million women of reproductive age in developing countries had an unmet need for contraception, a situation that has been ascribed to many factors, including cultural or religious opposition and gender-related barriers [11].

Similarly, although the maternal mortality ratio (MMR, number of maternal deaths per 100,000 live births) dropped by about 38% worldwide between 2000 and 2017, maternal and child health (MCH) inequities continue to persist, as many health systems continue to reflect and reinforce restrictive gender norms in society [12]. The World Health Organization’s (WHO) [13] strategies towards ending preventable maternal mortality include identifying and addressing barriers, including cultural beliefs and practices that prevent women from receiving or seeking care during pregnancy and childbirth at both the health system and societal levels, ensuring equal access to resources, education (including comprehensive sexuality education), focused efforts to eliminate gender-based violence and discrimination, including disrespect and abuse of women using health care services, and enabling women to exercise autonomy over their own reproductive lives and health care decisions [13].

A recent scoping review of maternal, newborn and child health in low- and middle-income countries revealed six key gendered dimensions of vulnerability and resilience: (1) restricted maternal access to financial and economic resources; (2) limited economic contribution of women as a result of motherhood; (3) social norms, ideologies, beliefs and perceptions inhibiting women’s access to maternal healthcare services; (4) restricted maternal agency and participation in reproductive decisions; (5) power dynamics and experience of intimate partner violence (IPV) contributing to adverse health outcomes for women, children and their families; and (6) partner emotional or affective support being crucial for maternal health (MH) and prenatal and postnatal wellbeing [14]. The emerging evidence also suggests that more gender-equitable attitudes among men and women and reduced gender inequalities in power in intimate relationships are associated with (a) a reduced likelihood of gender-based violence perpetration [6,15]; (b) increased willingness to engage in violence-preventive behaviors [16]; (c) greater discussion about household matters [17]; (d) reduced risk of sexually transmitted infections (STIs) and HIV acquisition [18]; increased negotiation of safer sex [19]; and current use of modern contraception [20].

Comparisons across studies have been limited by the lack of a single widely accepted definition of gender-equitable attitudes or of fixed components of a gender equity score [21,22]. Many studies have used adaptations of a widely tested scale, the Gender-equitable Men (GEM) scale [19], to measure attitudes towards gender norms in intimate relationships or differing social expectations for men and women and boys and girls. Few studies have examined how the negative consequences of gender-inequitable attitudes and behaviors on women’s access to health care and health outcomes might be compounded by poverty, ethnicity, and other forms of social disadvantage (that is, the intersectionality dimension of the social determinants of health [23,24]). For example, the literature suggests that in settings where women’s sexual behavior and contraceptive use are sanctioned only within marriage, young unmarried girls may delay care seeking due to shame stigma, and the fear of being judged by health care providers [4] (see also Svanemyr, 2020 [25]).

While there is widespread recognition that men, mothers-in-law, and older family members are often custodians of women’s access to health care [26], few studies have tested whether the influence of gender-equitable attitudes/behaviors among male partners is significant and distinct from the influence of social norms, specifically, women’s perceptions about what other people do and approve of. Furthermore, research on maternal and newborn health (MNH) outcomes is limited and empirical research has focused primarily on IPV perpetration and victimization, for which the influence of male power, gender ideology, and gender inequalities can be more clearly delineated, and on HIV and STIs. As Weber et al. [5] noted, existing data sets rarely contain both gender-related attitude data and health related data, an important shortcoming in the field, and many data sets are not powered to study how gender norms and related attitudes intersect with other measures of social disadvantage. In general, data gathered from male partners are lacking.

Using the Momentum project’s 2018 baseline survey and 2020 endline survey data of first-time mothers (FTMs) age 15–24 and their male partners in Kinshasa, the Democratic Republic of the Congo (DRC), this study examined the association between gender-equitable attitudes and behaviors among male partners and women’s postpartum family planning (PPFP)- and MNH-related outcomes. We analyzed how gender-equitable attitudes/behaviors among male partners intersected with women’s marital status to influence these outcomes. We hypothesized that gender-equitable attitudes and behaviors among male partners would have significantly greater influence on PPFP and MNH outcomes among unmarried than married adolescent/young FTMs as gendered social norms tend to be more enforced within marital unions. Although society may not hold boys and men accountable for their sexual behavior, reinforcing the notion that the responsibility for reproductive health falls solely on women, especially those who are unmarried [27], due to possible social norms against premarital sex, young unmarried mothers may be especially vulnerable to economic difficulties and health problems. This context may amplify the association between male partners’ gender-equitable attitudes and behaviors and our PPFP and MNH outcomes of interest among never married FTMs. A third objective was to examine the extent to which the influences of gender-equitable attitudes/behaviors among men were distinct and separate from relevant social norms about PPFP use and exclusive breastfeeding. The study extends the growing body of literature on how gender inequities and men’s endorsement of gender norms shape women’s health outcomes and intersect with their social identities. It is hoped that the findings will provide a more nuanced understanding of and shape future gender-transformative male involvement programs.

### Context

As the third most populous country in sub-Saharan Africa, the DRC has over 89.6 million inhabitants, 46% of whom reside in urban areas. The country has the third highest fertility rate in the world (5.9), is growing at a rate of almost three percent per year, and is one of the top four contributors to world population growth [28]. DRC is among the five poorest nations in the world. In 2018, about 73% of Congolese, about 60 million people, lived on less than $1.90 a day. The DRC ranked 175 out of 189 countries on the 2020 Human Capital Index, a reflection of prolonged conflict and fragility. The DRC’s Human Capital Index is 0.37, which is below the average of 0.4 for sub-Saharan Africa [29], and implies that Congolese children born today can expect to achieve only 37% of their potential. Congolese women face significant barriers to their empowerment in society and interpersonal relationships, with the DRC ranking 150 out of 163 countries on the gender inequality index, a measure reflecting gender-based disadvantage in three dimensions— reproductive health, empowerment and the labor market–in 2019 [29].

Against this backdrop, the modern contraceptive prevalence increased in the capital city of Kinshasa from 18.5% in 2013 to 26.7% in 2017 among married women [30]. However, unmet need for FP remains high. Estimates from the Population Reference Bureau [31] indicate that between 2007 and 2013, unmet need for FP among young married women decreased slightly from 32% to 30%. However, among sexually active unmarried women, unmet need increased from 38% to 45%. Qualitative studies have revealed that barriers to contraceptive use include the lack of male engagement; out-of-pocket copayment for contraceptives; reliance on amenorrhea for pregnancy prevention without knowing its limits; misconceptions about modern contraceptives; low prioritization of scheduled postpartum visits by women; and limited availability of readily accessible methods and PPFP counseling materials; sociocultural norms, especially the dominant position of the male in family decision making; pressure from family members to avoid modern contraception; and lack of information, misinformation, and disinformation [32,33].

Other qualitative studies in sub-Saharan African countries have noted that low partner involvement in and support for FP deter women’s contraceptive use (see for example, Akamike et al., 2020 [34]). Although male involvement in MCH is critical to the improvement of care-seeking behaviors and practices for women and children in low and middle-income areas, levels of male involvement in MH have remained low [35]). A major component of comprehensive antenatal care (ANC) is birth preparedness and complication readiness (BP/CR). However, as Mersha [36] highlighted, while strengthening BP/CR leads to a reduction in maternal mortality and morbidity, male involvement in BP/CR is quite poor throughout Sub-Saharan Africa.

In 2017, the MMR in the DRC was estimated at 473 per 100,000 live births [37], which was considerably higher than the global target of less than 70 per 100,000 live births by 2030. The adolescent birth rate has remained high and was estimated at 109 births per 1000 women aged 15–19 years in 2017–2018 [38], and short birth intervals (less than 24 months) are common (27%) [39]. Kinshasa has one of the highest institutional delivery rates in sub-Saharan Africa (more than 90%). The ANC coverage rate remained unchanged from 2010 to 2018 (95%), while skilled birth attendance increased from 96% to nearly 100% during the same period [40]. However, the quality of facility-based MNH care services remains poor [41].

Previous studies in the DRC have found high levels of agreement with gender-inequitable beliefs, such as “a man should have the final say in family matters” and “the woman’s primary role is caring for her home” among both men and women, especially men. Some components of the GEM scale have also received strong support from Congolese men (e.g., “men need sex more than women do”; “men don’t talk about sex, they just do it”; and “pregnancy prevention is women’s responsibility”) [42,43,44]. While some studies in the DRC have examined the extent to which social norms and/or gender-inequitable attitudes influence contraceptive use intentions and other health and social outcomes such as IPV, analyses have been conducted on men and women separately [43,45], on women only [46] or men only [44]. When studies have examined correlates of fertility-, FP-, or IPV-related outcomes in the DRC and other sub-Saharan African countries, the gender attitudes of the spouse/partner have rarely been included as a predictor of the women’s health outcomes.

Yet, the evidence demonstrates that improvements in gender-related factors such as gender-equitable attitudes of men can result in positive FP and MH outcomes [47,48,49]. For example, more gender-equitable attitudes among men were found to be associated with partner communication about condom use [50] and use of modern contraceptives [51] in Kenya and Ethiopia. A study in Tanzania found that more gender-equitable attitudes among women were significantly and positively associated with women’s reports of contraceptive use; however, husbands’ gender-equitable attitudes were not [52]. In such rare instances in which men’s gender equitable attitudes have been included as predictors of women’s health outcomes, behavioral indicators of gender equity among men have been rarely considered. For example, although Nanda et al. [52] examined the association between women’s role in household decision making and contraceptive use, their indicator was not based on actual patterns of decision making but on the husband’s and wife’s beliefs about who should have the greater say in specific decisions. These studies highlight the need to strengthen the evidence from couple studies on the influence of gender relations on FP and MH outcomes.

## 2. Materials and Methods

### 2.1. Data

The analysis was based on secondary data from the Momentum baseline and endline surveys of FTMs age 15–24 and their male partners. Momentum was a quasi-experimental community-based pilot project conducted in three intervention health zones (Kingasani, Lemba, and Matete) and three comparison health zones (Bumbu, Masina I, and Ndjili) of Kinshasa. Project objectives were to increase PPFP uptake and the adoption of care seeking behaviors and household practices beneficial to mother and baby, and to promote gender-equitable attitudes and behaviors. Home visits and support group education were provided to FTMs and their male partners by 75 male and 75 female trained third-year students from 11 nursing schools (Institut Technique Médical). Home visits included gender-integrated counseling on FP, MNH and nutrition, PPFP distribution, simple health checks for mother and baby, treatment of common childhood illnesses, and referrals. FTMs were enrolled at six months’ gestation and followed up for sixteen months.

Using pre-tested questionnaires, trained data collectors interviewed FTMs and their male partners at baseline (September-November 2018) and endline (May-August 2020). FTMs and male partners were asked questions on the following topics: (a) household characteristics; (b) background characteristics; (c) reproductive history; (d) use of FP methods; (e) ANC; (f) delivery, postnatal care, and newborn care; (g) fertility preferences; (h) gender-relations; and (j) exposure to the Momentum interventions. In addition, male partners were asked questions on IPV perpetration. Data were collected via smartphones using the SurveyCTO mobile data collection application. A unique quick response code assigned at baseline to the couple (FTM and male partner) permitted participants’ endline data to be linked to their baseline data. Our analytic sample consisted of 1335 FTMs 15–24 and their male partners who were successfully interviewed in the baseline and endline surveys, whose data could be linked, and who had no missing data on variables included in the analysis. For the MNH outcomes, the analytic sample was restricted to FTMs with live births. See Table S1 for the percent distribution of FTMs by loss to follow-up status and baseline socioeconomic characteristics.

### 2.2. Variables

#### 2.2.1. Outcomes

All outcomes were measured using the FTM endline data set. There were four binary PPFP-related outcomes: (1) discussion of FP with the male partner in the immediate postpartum period (that is the first six weeks following childbirth/pregnancy loss); (2) obtaining a contraceptive method from a health facility, pharmacy, or store, in the immediate postpartum period; (3) modern contraceptive use 0–2 months after childbirth/pregnancy loss; and (4) modern contraceptive use within 12 months of childbirth/pregnancy loss.

MNH-related outcomes were:

- FTM-reported shared MNH decision making: This composite index measured the number of MNH-related decisions that the FTM made jointly with her husband/male partner, such as when to start seeking ANC and how soon to start breastfeeding (see the full list of items in Table S2). The FTM-reported shared MNH decision-making index consisted of nine items and had a Cronbach’s alpha of 0.841 at endline. Analysis of this outcome was restricted to FTMs with live births.
- Completion of the MH continuum of care: This binary variable measured completion of all three sequential recommended maternal healthcare services: four or more ANC visits, at least one of which was with a health professional; delivery in a health facility; and postpartum check within 48 h of delivery. Analysis of this outcome was restricted to FTMs with a live birth. Although the 2016 WHO Guidelines on Antenatal Care for a Positive Pregnancy Experience recommended at least eight ANC visits to reduce perinatal mortality and improve women’s experience of care [53], at the time of the study, the DRC Ministry of Health guidelines on the number of ANC visits had not been updated to reflect the new standards. Therefore, we used four or more ANC visits to reflect adequate ANC.
- Exclusive breastfeeding (EBF) for six months: FTMs were coded “1” if they reported that their baby received only breastmilk until 6 months of age and no other liquids or solids, not even water (except for oral rehydration solution or drops/syrups of vitamins, minerals, or medicine), and “0”, otherwise. Analysis of this outcome was restricted to FTMs with a live birth that survived for 6 or more months.

#### 2.2.2. Gender-Equitable Attitudes and Behaviors among Male Partners

Four measures were constructed, three of which were derived from the male partners’ baseline data:

- GEM Scale. The GEM scale is a measure of attitudes towards gender equality or separate roles for men and women, and previous psychometric testing has yielded satisfactory validity and reliability [18,19], including for the DRC sample in a multi-country study of men’s lifetime perpetration of physical IPV [44]. In the present study, the GEM scale was constructed from 11 statements covering gender norms, violence, sexuality, masculinity, and reproductive health, with response codes “totally agree”, “partially agree”, or “disagree.” Items are listed in Table S3. Responses to some of the statements were reverse-coded to reflect gender-equitable attitudes. Item analysis and factor analysis with rotation were used to test the construct validity of the GEM scale and clarify its domains. Factors loading less than 0.3 were dropped and the GEM scale score was constructed from the predicted values of the first factor which had an eigen value greater than 1. The components of this score had a Cronbach’s alpha of 0.722, with higher values of the score representing more supportive attitudes towards gender equity.
- History of IPV perpetration: At baseline, male partners of FTMs were asked whether they had ever perpetrated 13 acts of physical, sexual, and emotional violence against the FTM. The acts of violence included in the questionnaire were adapted from the Conflict Tactics Scale used in DRC’s most recent Demographic and Health Survey [54]. An affirmative response to any of these acts indicated that the male partner had a history of IPV perpetration in his relationship with the FTM.
- MH involvement index: This additive index was constructed from the male partner’s endline survey data and included the following binary items/actions measuring the male partner’s presence at the various occasions during pregnancy and labor, and his involvement in ANC and birth planning (e.g., sitting in the consultation room with the FTM during the checkup, etc.). As FTMs were asked the same questions, missing data on any of the male partner-reported items were replaced with the FTM’s reports of his involvement in that activity. This 12-item index ranged from 0 to 12, had a Cronbach’s alpha (scale reliability coefficient) of 0.851 at endline and a KMO coefficient of 0.879. See Table S4 for descriptive statistics of the individual components of the index.
- Index of willingness to perform caregiving activities for infants: In the baseline survey, male partners were asked how willing they were to perform 11 caregiving activities after their baby was born (e.g., changing the baby’s diapers, etc.). Response categories were “not at all willing”, “somewhat unwilling”, “undecided”, “somewhat willing”, or “extremely willing.” The response for each component was coded as 1 if the male partner was extremely willing to perform the activity, and 0 otherwise. Cronbach’s alpha for the 11 items was 0.916. Factor analysis revealed a distinct one-factor solution, with an eigen value of 4.004 and all components had factor loadings exceeding 0.45. The index of willingness was constructed from the predicted values of the first factor, with higher values of the index representing greater willingness of the male partner to engage in infant caregiving activities. See Table S5 for the descriptive statistics of the individual components of the index.
- Male-partner-reported shared MNH decision making: This composite index was included in the regressions of completion of the maternal continuum of care and EBF for six months. This index was created in the same way as the FTM-reported index but was based on the male partner’s report. The male-partner shared MNH decision making index consisted of 9 items and had a Cronbach’s alpha of 0.804 at endline.

#### 2.2.3. Control Variables for PPFP Outcomes

The following control variables were specific to the PPFP analysis and were based on the FTMs’ data: pre-pregnancy use of FP, partner discussion of FP in the immediate postpartum period (included in the analyses of obtaining a contraceptive method from a health facility, pharmacy, or store in the immediate postpartum period, and modern contraceptive use in the immediate and extended postpartum periods). Guided by the Integrated Behavioral Model, we also controlled for the FTM’s attitudes toward and knowledge of FP at baseline (whether she approved of FP use in the six weeks following childbirth/pregnancy loss, the number of FP myths and misconceptions that she rejected, and the number of modern FP methods known), as well as for the FTM’s baseline perceptions of PPFP norms. Questions about FP myths and misconceptions asked FTMs the extent to which they strongly agreed, agreed, disagreed, or strongly disagreed, coded “1”, “2”, “3”, and “4”, respectively) with eight FP myths and misconceptions (e.g., “Contraceptives are dangerous to women’s health”, etc.). The index was constructed as the sum of the “strongly disagree” responses with higher values representing greater rejection of FP myths (range: 0 to 8; α = 0.865).

Injunctive norms measured the FTM’s belief about whether most referents approved or disapproved of FP use in the immediate postpartum period. The index was measured by first asking the participant to list up to five people who were most important to her, either generally, or when deciding about the use of a method of contraception and whether each of those referents would approve or disapprove of the FTM’s use of FP in the immediate postpartum period. The FTM’s belief as to whether a given referent approved or disapproved of her use of PPFP in the immediate postpartum period was weighted by her motivation to do what each referent thought. The injunctive norm index ranged from 0 to 20.

Descriptive norm was a binary variable that measured the FTM’s baseline belief about whether more than half of or all FTMs age 15–24 years in her community used contraceptive methods in the immediate postpartum period. Normative expectations about PPFP measured whether the FTM believed that most people who were important to her thought that she ought to (a) discuss use of a method of contraception in the immediate postpartum period with her husband/partner before the baby was born; (b) start using a method of contraception in the immediate postpartum period, (c) even if she was breastfeeding her baby, and thought that (d) women have a right to make FP decisions. Responses (strongly agree, agree, disagree, and strongly disagree) were reverse-coded, and an additive scale created, with higher values representing greater perceived normative expectations about PPFP use. The scale reliability coefficient for the resulting index was 0.825. Perceived community approval of PPFP use consisted of three categories reflecting the FTM belief that community members would say “good things” (reference group) or “bad things” about women who used FP in the immediate postpartum period or whether community members would be indifferent.

To measure FTM’s personal agency at baseline, we constructed a seven-item summative index of PPFP-related self-efficacy from questions about the level of confidence the FTM had in her ability to perform seven behaviors: (a) discuss use of a method of contraception in the immediate postpartum period with her husband/partner; use a method of contraception in the immediate postpartum period even if she was afraid that her husband/partner would (b) get angry at her (c) reject her; (d) think she was having sex with someone else; and (e) stop giving her money for food and other necessities, (f) go to a health facility, pharmacy or store to ask for/buy a method of contraception in the immediate postpartum period, without feeling embarrassed, and (g) refrain from having sex if she and her husband/partner were getting “turned on” in the immediate postpartum period and she could not bring up the subject of protection. Responses were on a four-point Likert scale: not at all confident (1); not confident (2); confident (3); and extremely confident. The index had a Cronbach’s alpha of 0.921 and ranged from 7 to 28. The higher the index, the greater was the FTM’s level of PPFP self-efficacy at baseline.

#### 2.2.4. Control Variables for MNH Outcomes

Our analysis of the FTM’s completion of the maternal continuum of care included two measures of ANC content based on women’s self-reports: (a) an adapted index of WHO-recommended actions for a positive pregnancy experience [53]; and (b) an adapted index of ANC counseling for health promotion and disease prevention [55]. The adapted index of WHO-recommended actions for a positive pregnancy experience measured whether the FTM received or experienced the following elements at any point during her pregnancy: weighed, abdomen felt, blood pressure measured, urine sample taken, blood sample taken, iron tablets or syrup given, drugs against malaria prophylaxis (sulfadoxine-pyrimethamine/fansidar) tablets given, provider listened to baby’s heartbeat. Factor analysis yielded a distinct one-factor solution (eigen value = 1.143). An eight-item additive index was constructed with a scale reliability coefficient of 0.549 at endline. See Table S6 for descriptive statistics of the individual components of the index.

The adapted index of ANC counseling for health promotion and disease prevention measured whether the FTM received ANC counseling on 11 topics: (a) EBF; (b) newborn care; sleeping under insecticide-treated net; (c) birth preparedness; (d) delivery with skilled birth attendant; (e) birth spacing; (f) FP; (g) prevention of mother-to-child transmission of HIV; (h) foods the FTM should eat; (i) obstetric danger signs; and (j) newborn danger signs. The resulting additive index had a Cronbach’s alpha of 0.823 and ranged from zero to 11. See Table S7 for descriptive statistics of the individual components of the index.

The analysis of EBF for 6 months did not include these ANC indices but controlled for the FTM’s receipt of ANC counseling on EBF (no, yes). The choice of other explanatory variables for the EBF regressions was guided by the Integrated Behavioral Model and included the following baseline characteristics of the FTM: (a) perceived EBF norms and (b) EBF autonomy, a measure of personal agency. To measure injunctive EBF norms, the FTM was first asked to list up to five people who were most important to her either generally, or when deciding how to take care of her baby and to specify their relationship to her. Next, the FTM was asked whether each referent would approve or disapprove of her exclusively breastfeeding her baby. The injunctive norms index was constructed by weighting the responses about each referent’s perceived approval of EBF with the FTM’s motivation to comply, which was based on the following question: “Please tell me whether you strongly agree, agree, disagree, or strongly disagree with each of the following statement: When it comes to breastfeeding my baby, I want to do what [REFERENT] thinks I should do.” The injunctive norm scale ranged from 0 to 20.

EBF descriptive norms were measured by asking participants how many of the women who were important to them practice/have practiced EBF: all of them, more than half of them, about half of them, less than half of them, or none of them. The response categories “all of them” and “more than half of them” were combined and coded as “1”, with the remaining categories being coded as “0.” Normative expectations about EBF measured whether the FTM strongly agreed that most people who were important to her thought she ought to exclusively breastfeed her baby. EBF autonomy, a proxy for freedom from external control or influence in EBF decisions, was based on an affirmative response to the following question: “If most of the people who are important to you did not want you to practice EBF, would you still do it?”

#### 2.2.5. Control Variables for All Outcomes

All PPFP and MNH regressions controlled for the following baseline characteristics of the FTM: unintended pregnancy, residence in the intervention health zones, age, being never married, employment in the past 12 months, years of schooling, Bakongo ethnicity, and household wealth. Household wealth reflected tiers (low, medium, and high) of an index constructed from the floor, wall, and roofing materials of the dwelling, availability of electricity, use of improved drinking water sources, type of toilet, and ownership of household items (radio, television, telephone, computer, refrigerator, stove, watch, mobile phone, bicycle, motorcycle, animal-drawn cart, car, and a boat with a motor). The index was constructed from principal components analysis, consisted of 19 items, and had a Cronbach’s alpha of 0.642.

### 2.3. Statistical Analysis

Descriptive analysis was used to summarize the characteristics of the sample and the results presented as percentages or means. Bivariate associations were analyzed of the prevalence/means of the PPFP and MNH outcomes by variables measuring male partners’ gender-equitable attitudes and behaviors. For this stage of the analysis, we performed a median split on continuous measures of gender-equitable attitudes and behaviors. Next, we ran multivariable logit models for all outcomes except the FTM-reported index of shared MNH decision making, for which ordinary least squares regression was used. Results were presented as adjusted odds ratios (AOR) or adjusted coefficients and 95% confidence intervals (CI). A *p*-value of 0.05 was used as the cut-off point for significant association.

Two models were run for each PPFP outcome. Model 1 was the model with the full set of explanatory variables. Given that marital status might have an impact on the extent to which male partners’ characteristics influence FTMs’ PPFP or MNH outcomes, the multivariable analysis was first stratified by marital status (never married versus ever married/formally engaged). Model 2 then included interaction terms between variables measuring gender-equitable attitudes and behaviors among male partners and the FTM’s marital status. For each MNH outcome, three models were run. Model 1 included only measures of the male partner’s gender-equitable attitudes and behavior, Model 2 was the full model, and Model 3 added the interactions between the male partner’s gender-equitable attitudes and behavior and the FTM’s marital status. The analysis was conducted using Stata 17 [56].

### 2.4. Ethical and Country Approval

The study received IRB approval from the Tulane University Biomedical Institutional Review Board (2018-1028) and the University of Kinshasa School of Public Health Ethics Committee (ESP/CE/066/2018). Approval was granted for data collection among FTMs and male partners age 15–17 years without parental/guardian consent because some were legally married and all were undertaking adult responsibilities. Written informed consent was provided by all survey participants on paper forms and via smartphone prior to data collection (both surveys). At baseline, FTMs also provided written consent for their husband/male partner to be involved in the Momentum study.

### 2.5. Patient and Public Involvement

Patients and the public were not involved in the study design, development of the research questions, recruitment into or implementation of the study, or the definition of the outcome. Results were not distributed to the participants themselves.

## 3. Results

### 3.1. Sample Characteristics

Table 1 summarizes the characteristics of FTMs and their male partners. At baseline, slightly more than half of the male partners had a history of IPV perpetration. GEM scores were low and ranged from −0.02 to −0.01. Male partners were involved in an average of seven out of 13 MH activities and participated in an average of 1.7 shared MNH decisions (range: 0–9). Their level of willingness to perform caregiving activities for infants was low. The mean willingness index scores varied from 0.035 (for the PPFP analytical sample) to 0.68 (for the EBF analytical sample).

At baseline, over half of the FTMs were 20–24 years, about 20% were never married, a quarter were unemployed and over a third identified their ethnicity as Bakongo. FTMs had on average 11 years of schooling (range: 0–18) and less than half lived in the intervention health zones. Although FTMs’ PPFP attitude and knowledge were moderate, their unintended pregnancy rate was high (about 80%). Seventy-four percent of FTMs approved of PPFP use and they knew on average six modern methods (range: 0–11). The mean FP myth rejection index, which ranged from 8 to 32, was 19.8 and the mean injunctive norms index was 10.3 (range: 0–30). Approximately 12% of FTMs believed that more than half of the FTMs in their community used FP and only a third believed that the community would say good things about women who used FP in the immediate postpartum period. The mean normative expectation index was relatively high (mean: 11.0, range: 4–16), suggesting that normative expectations were supportive of PPFP use.

Slightly more than half of the FTMs had used a FP method before their pregnancy and reported they had discussed FP with their partner in the immediate postpartum period. The mean self-efficacy index was 19.0 out of a maximum of 28. Nine out of 10 FTMs were counseled on EBF during ANC visits. The mean indices for WHO-recommended actions for a positive pregnancy and ANC counseling were high, with average scores of 7.4 (range: 0–8) and 9.2 (range: 0–11), respectively.

Autonomy in decision making about EBF was high among the surveyed FTMs. About three in five FTMs reported they would practice EBF against the wishes of people important to them. However, descriptive norms and normative expectations were not supportive of EBF. Sixteen percent of FTMs believed more than half of women who were important to them practiced EBF and three in ten agreed that people important to them thought they ought to exclusively breastfeed their baby. The mean EBF injunctive norms index was 9.7 (range 0–20).

### 3.2. Bivariate Results

#### 3.2.1. PPFP Outcomes

As shown in Table 2, 56% of FTMs discussed FP with their partner in the immediate postpartum period and a quarter obtained a FP method during that period. Only nine percent of FTMs had used a modern FP method in the immediate postpartum period; however, within 12 months of childbirth and pregnancy loss, 47% of FTMs reported using a modern FP method. The prevalence of the PPFP outcomes examined did not vary by level of the GEM scale, history of IPV perpetration, and the degree of willingness to perform caregiving activities for infants. However, a male partner’s level of MH involvement was significantly associated with the prevalence of each PPFP outcome, except use of a modern FP method in the immediate postpartum period. The percentage of FTMs who reported they discussed FP with their partner, obtained a FP method in the immediate postpartum period and used a modern FP method within 12 months of childbirth or pregnancy loss was greater if the male partners was highly involved in MH activities.

#### 3.2.2. MNH Outcomes

Almost three-quarters of FTMs had completed the MH continuum of care and two in five exclusively breastfed their newborn for six months (Table 3). Reports of shared MNH decision making were greater among FTMs than among their male partners. FTMs reported participating in an average of 3.0 shared decisions (SD: 2.7), while male partners reported participating in 1.7 shared decisions (SD: 2.4), as was shown in Table 1. There was little or no difference in the percentage of FTMs who completed the MH continuum of care and exclusively breastfed for six months by level of the GEM scale, history of IPV perpetration, and willingness to perform caregiving activities for infants. The MH continuum-of-care completion rate increased significantly from 69% among FTMs whose male partners had low MH involvement indices to 75% among those with male partners who had high indices. Likewise, the percentage of FTMs who exclusively breastfed for six months increased with male partner involvement in MH from 29% to 45% among those in the low and high categories, respectively.

Both gender-equitable attitudes and MH involvement were significantly associated with the mean shared MNH decision making index calculated from FTMs’ reports. For instance, the mean shared MNH decision making index was 2.1 (SD: 2.4) among FTMs with male partners who had low gender-equitable attitudes while those with male partners who had high gender-equitable attitudes participated in an average of 3.1 joint MNH decisions (SD: 2.8). No significant differentials in the MNH outcomes were observed by history of IPV perpetration and degree of willingness to perform caregiving activities for infants.

### 3.3. Multivariate Results

#### 3.3.1. PPFP Outcomes

Table 4 presents the regression results for PPFP behaviors after controlling for the FTMs’ baseline characteristics. None of our measures of the male partners’ gender-equitable attitudes and behaviors were significantly associated with FTMs’ PPFP outcomes, except for partner discussion of FP. For this outcome, the results indicated that the higher the index of male involvement in MH activities, the greater were the odds of discussion of FP with the partner in the immediate postpartum period (AOR = 1.18, 95% CI = 1.13, 1.23).

Several indicators of the FTM’s PPFP attitude, knowledge and perceived norms were significantly associated with PPFP behaviors. FTMs had significant and higher odds of obtaining a FP method, using a modern FP method in the immediate postpartum period and within 12 months of childbirth or pregnancy loss if they rejected more FP myth/misconceptions. A unit increase in the number of FP myths/misconceptions rejected was associated with 1.05, 1.06 and 1.03 increase in the odds of obtaining a FP method and using a modern FP method in the immediate postpartum period, and with modern contraceptive use within 12 months of childbirth or pregnancy loss, respectively. Knowledge of modern FP methods was significantly associated with discussing FP with a partner in the immediate postpartum period (AOR = 1.07, 95% CI = 1.01, 1.13). The injunctive norms index was positively associated with obtaining a FP method in the immediate postpartum period (AOR = 1.04, 95% CI = 1.01, 1.08) and use of a modern contraceptive method within 12 months of childbirth or pregnancy loss (AOR = 1.03, 95% CI = 1.00, 1.05). FTMs who perceived that the community would be indifferent to their use of PPFP were less likely to obtain a FP method in the immediate postpartum period (AOR = 0.45, 95% CI = 0.31, 0.66) and use a modern contraceptive method within 12 months of childbirth or pregnancy loss (AOR = 0.74, 95% CI = 0.55, 0.99).

Unintended pregnancy and work in the past 12 months were not significantly associated with any of the PPFP behaviors. The odds of discussing FP increased with age (AOR = 1.1, 95% CI = 1.00, 1.11), while never married FTMs had significantly lower odds of using a modern FP method within 12 months of childbirth or pregnancy loss than their counterparts who were ever married or formally engaged (AOR = 0.60, 95% CI = 0.45, 0.80). As expected, FTMs in the intervention health zones had higher odds of performing each PPFP behavior than those in the comparison health zones. Previous use of FP (i.e., before the pregnancy) was a significant predictor of FP use within 12 months of childbirth or pregnancy loss (AOR = 1.36, 95% CI = 1.07, 1.73), and partner discussion of FP in the immediate postpartum period was also a positive predictor of obtaining a FP method in the immediate postpartum period (AOR = 5.46, 95% CI = 3.90, 7.66) and using a modern contraceptive method within 12 months of childbirth or pregnancy loss (AOR = 1.54, 95% CI = 1.21, 1.95).

Interaction terms between marital status and (1) gender-equitable attitudes, (2) history of IPV perpetration, (3) MH male involvement and (4) willingness to perform caregiving activities for infants were added to the PPFP regressions and results are shown in Table 5. The male partner’s gender-equitable attitudes had significantly more positive associations with the odds of obtaining and using a FP method in the immediate postpartum period among never married FTMs than among those who were ever married or formally engaged. The male partner’s history of IPV perpetration had a significantly more negative association with obtaining a FP method in the immediate postpartum period (AOR = 0.42, 95% CI = 0.21, 0.85), but a more positive association with use of a modern method of within 12 months of childbirth or pregnancy loss (AOR = 1.83, 95% CI = 1.01, 3.30) among never married FTMs than among those who were ever married or formally engaged. The male partner’s willingness to perform caregiving activities for infants had a significantly more positive association with discussion of FP in the immediate postpartum period and use of a modern contraceptive method within 12 months of childbirth/pregnancy loss among never married FTMs than among their counterparts who were ever married or formally engaged.

#### 3.3.2. MNH Outcomes

Table 6, Table 7 and Table 8 present regression results for the MNH outcomes. Model 1 includes only measures of gender-equitable attitudes and behaviors among male partners. Model 2 adds control variables. Model 3 adds interaction terms between the male partners’ gender-equitable attitudes and behaviors and marital status, and in Table 7 and Table 8, Model 4 adds an interaction term between the number of shared MNH decisions reported by the male partner and marital status. As shown in Table 6, Table 7 and Table 8, some significant associations were found between gender-equitable attitudes and behaviors among male partners and two of the MNH outcomes explored, after controlling for baseline characteristics of the FTM. With each increase in the male partner’s MH involvement index, the index of shared MNH decisions reported by the FTM increased (adj β = 0.24, 95% CI = 0.17, 0.27), as did the odds of EBF for six months (AOR = 1.11, 95% CI = 1.05, 1.16). The number of shared MNH decisions reported by the male partner was also positively associated with EBF for six months, whereby the higher the number of shared MNH decisions, the greater were the odds of EBF (AOR = 1.08, 95% CI = 1.02, 1.14). None of the male partner’s gender-equitable attitudes and behaviors were associated with the FTM’s completion of the MH continuum of care. Interactions between marital status and the selected predictor variables were also tested but none were statistically significant.

Regarding FTMs’ baseline characteristics, Table 6, Table 7 and Table 8 show that work in the past 12 months, marital status, education, household wealth and ethnicity were not significantly associated with any MNH outcome. FTMs who had unintended pregnancies had significantly lower shared MNH decision making indices than those whose pregnancies were intended and FTMs age 20–24 years had significantly higher shared MNH decision making indices than those age 15–19. Surprisingly, FTMs living in the intervention health zones had significantly lower odds of completing the MH continuum of care than those living in comparison health zones. The results also showed that the index of WHO-recommended actions for positive pregnancy (AOR = 1.55, 95% CI = 1.32, 1.82) and the index of ANC counseling (AOR = 1.13, 95% CI = 1.05, 1.21) had significant positive associations with the odds of completing the MH continuum of care. EBF autonomy and receipt of ANC counseling on EBF (AOR = 2.33, 95% CI = 1.27, 4.27) were associated with significantly higher odds of EBF.

We also ran regression models to identify predictors of shared MNH decision making as reported by male partners. The low degree of concordance between male partners’ and FTMs’ reports of joint decision making and the results of regression models of the index of shared MNH decision making that was derived from male partners’ reports are shown in Table S8 and Table S9, respectively. The male partner’s gender-equitable attitude was a significant positive predictor of the male partner-reported shared decision-making index after controlling for other factors.

## 4. Discussion

This is one of few studies (that we are aware of) to investigate multiple measures of gender-equitable attitudes and behaviors among male partners and to assess the strength of their association with PPFP and MNH outcomes among adolescent/young first-time mothers in sub-Saharan Africa. An important strength of the study is the examination of intersectionality between male partners’ gender-equitable attitudes and behaviors and FTMs’ marital status. This examination is important due to the adverse psychological and social consequences of pregnancy and childbirth for unmarried young women, which tend to be more severe than the consequences for unmarried young men. These consequences could include social stigma for unmarried mothers and their children, limited education, a heavy economic burden, depression, and loss of confidence [57,58].

Our study measured various dimensions of gender equity among male partners. At the individual and attitudinal levels, the GEM scale captured endorsement of gender norms, and we also measured the male partner’s willingness to participate in routine childcare activities. At the couple level, we measured male partner-reported gender equality in decision making about MH issues and male partner involvement in MH, which captured partner support in pregnancy. The gender-based power dimension was captured at baseline by the male partner’s lifetime perpetration of IPV against the FTM. Based on the results of our analysis, we conclude that there was insufficient evidence to identify one particular measure of gender-equitable attitudes/behavior among male partners that was an overwhelmingly significant predictor of PPFP and MNH outcomes, after controlling for potential confounders. The GEM scale was positively associated with shared decision making about MNH issues, but only for the index that was based on male partners’ reports. The male partner’s involvement in MNH was positively associated with FP discussion in the immediate postpartum period and shared decision making about MNH issues. The male partner’s willingness to be involved in routine childcare activities and shared MNH decision making were both positively associated with the odds of EBF, and these associations were not moderated by the FTM’s marital status.

It is difficult to disentangle male involvement in MH and the role of gender norms and gender structures. Gender norms, beliefs, and expectations typically hinder male involvement in reproductive healthcare as well as their partners’ access to programs and services [59]. Gender structures strongly influence individual men’s decisions (including the sexual division of labor, power, and emotional and symbolic relations [59]). While a man’s perception of his role during and after childbirth can determine if he will allow his partner to access health services and if he will accompany her to ANC and delivery care sessions [59], in some instances, a woman’s acceptance into a healthcare facility is dependent on whether or not she is accompanied by a spouse. In sub-Saharan Africa, there has been a history of health providers denying services to women that come without a partner [60] and fast-tracking women who arrive with a partner for ANC services.

None of our male partner-related gender-equity measures were associated with FTMs’ odds of completing the MH continuum of care. Predictors of this outcome were the adapted index of WHO-recommended actions for a positive pregnancy experience and the adapted index of ANC counseling. Receipt of ANC counseling on EBF and the FTM’s EBF-related personal agency were significant predictors of the odds of practicing EBF for six months. Furthermore, the study revealed that injunctive norms exerted an independent influence on three of our four PPFP outcomes. The lack of significance of male partners’ gender-equitable attitudes and behaviors as predictors of the FTM’s completion of the MH continuum of care may be partly explained by the fact that men may generally feel disengaged and out of place at maternal healthcare services; and may be physically distanced outside the clinic. Even though men tend to be the primary decision makers for women’s access to care, prevailing sociocultural norms defining pregnancy and childbirth as women’s spaces may leave men disempowered and spatially and socially marginalized in maternal healthcare settings [61]. However, the low concordance between FTMs and male partners reports of joint decision making suggests that further research is needed to better understand effective couple communication and couples’ interpretation of each other’s role in the decision-making process.

Our results showed some important differences between never married and ever-married/formally engaged FTMs with respect to the significance of gender-equity measures for predicting PPFP outcomes. As the meaning of gender equity among male partners, particularly as it relates to fertility, may depend on a woman’s life course stage, different measures may need to be developed specifically for adolescents who are married and those who are unmarried to better understand the pathways between gender and positive FP outcomes. Mandal et al. [49] make similar arguments for measures of women’s empowerment.

Our results partially align with a few studies that indicate some male gender-equitable attitudes and beliefs about FP do not have a positive association with increased spousal communication and joint decision making about FP [62]. However, some of our findings support those of other studies. Evidence from sub-Saharan Africa indicates that positive changes in gender-equitable attitudes, joint healthcare decision making, and increased spousal communication were associated with increased relative probability of modern contraceptive use [63,64,65]. However, these studies also suggested that more research needs to be conducted on gender-equitable attitudes among males and their subsequent impact on contraceptive uptake. A qualitative study conducted in Nepal among men and women ages 15–24, showed that decision-making and preferences about FP methods among married participants were largely controlled by male partners, and not only were they reluctant to use FP methods, women also did not expect them to [66]. However, the same decision among unmarried participants warranted a discussion and was ultimately a joint decision [66]. Apart from this study, there is a general lack of data that specifically looks at the effect of marital status on FP and fertility decision-making.

### 4.1. Limitations

The limitations of our study warrant discussion. First, social desirability bias is a possible limitation as our study was based on self-reports and the potential existed for male partners to inflate their willingness to participate in routine child activities and endorsement of gender-equitable norms and underreport their perpetration of IPV. As most of the control variables and gender-related variables were measured at baseline and our outcomes were measured at endline, we could establish temporal sequence for our regression analysis to a large extent; however, our ability to establish causality was limited. Another limitation was the lack of generalizability and potential selection bias associated with the use of a convenience sample of FTMs and their male partners. Thus, the results may not be generalizable to FTMs who were 25 and older or other samples of adolescent/young FTMs and their male partners in Kinshasa.

In addition, our measures of male involvement in MH and willingness to participate in routine childcare activities were not validated. Given that gender inequality operates at the household, service-delivery, community and societal levels, there is a need to develop measures at these higher levels and adopt multilevel approaches to analyzing and addressing gender inequality. The low concordance between FTMs’ and their male partners’ reports of shared MNH decision making was a limitation of the study and calls for further research to better understand effective couple communication and couple’s understanding of each other’s role in decision making. Another concern was that several of the FTMs and male partners enrolled at baseline were lost-to follow-up (21% and 29%, respectively), which led to a smaller analytic sample size. During the endline interview, we attempted to mitigate the loss to follow-up by tracking down, whenever possible, study participants interviewed at baseline. As Table S1 showed, loss to follow-up was not random. Differences in socioeconomic characteristics between FTMs and male partners who continued to participate in the study and those who did not should be borne in mind when interpreting the results.

Despite these limitations, the results of our study provided a better understanding of how gender-related attitudes and behaviors among male partners were associated with PPFP and MNH outcomes among adolescent/young FTMs. Given the DRC’s high adolescent birth rate, high unmet need for FP, high MMR, and high poverty rate, the findings are germane to current efforts to promote healthy timing and spacing of pregnancy among adolescent and young mothers. We addressed a significant gap in the literature about how male engagement in MH and gender-inequitable attitudes among male partners impact EBF. Most studies have focused on HIV-positive mothers [67] and few have examined the impact of male decision making on the initiation, maintenance, and sustainment of EBF [68]. As gender norms are equally as important as gender-equitable attitudes, future research should incorporate measures of gender norms to further track progress towards the SDGs (5 and 10) and deepen our understanding of the influence of gender norms on FTMs’ PPFP and MNH behavioral outcomes.

### 4.2. Program Implications

The results of this study suggest that on a broader scale, programs need to address social norms that define what it means to be a man and that constrain men in becoming actively involved in MH and caregiving activities for infants. There is a need to identify targeted, more effective strategies/approaches to strengthen gender-equitable attitudes among men, especially those who are married. It is important for the health sector to be aware of the vital role that it plays in fostering women’s participation in decisions about their own health and EBF for six months through the active engagement of men in MH care. As gender norms tend to be entrenched, learned, and internalized throughout childhood and adulthood, it is important to encourage couples to model gender-equitable attitudes and behaviors in their relationship and transmit these values to their children. On a much larger scale, programs should encourage men who hold gender-equitable attitudes and practice gender-equitable behaviors in their own lives to become agents of attitudinal and behavior change, and mobilize communities, especially men, in participatory dialogue to accept women as equal partners in decision making on birth spacing and MNH.

## 5. Conclusions

This study has expanded our understanding of how gender-equitable attitudes and behavior among male partners may shape adolescent/young mothers’ PPFP and MH outcomes. Our results suggested that PPFP behaviors were shaped by the intersection of marital status with gender-equitable attitudes and behaviors among male partners to constitute advantage for some outcomes and result in greater vulnerability for others. Interventions aimed at fostering gender equitable and nonviolent attitudes and behaviors among men must use multiple measures to better understand how improvements in gender-related factors can lead to better health for young mothers and their babies.

## Figures and Tables

**Table 1:** Characteristics of the sample by outcome examined, first-time mothers age 15–24, Kinshasa, DRC.

Baseline Characteristics	Postpartum Family Planning	FTM-reported Shared MNH Decision Making	Completion of the MH Continuum of Care	Exclusive Breastfeeding
**Male partner’s gender-equitable attitudes and behaviors**				
GEM Scale ^1^	−0.015 (0.860)	−0.0122 (0.859)	−0.019 (0.854)	−0.01 (0.85)
History of IPV perpetration: %	51.5	51.8	51.4	52.1
MH involvement index ^1^	6.951 (3.035)	6.955 (3.024)	7.062 (2.944)	7.022 (2.930)
Index of willingness to perform caregiving activities for infants ^1^	0.035 (0.934)	0.037 (0.916)	0.058 (0.903)	0.068 (0.891)
No. of shared MNH decisions reported by male partner (a) ^1^	1.667 (2.424)	na	1.668 (2.430)	1.643 (2.405)
**FTM’s baseline characteristics**				
Unintended pregnancy: %	80.5	80.7	80.1	80.7
Intervention health zone: %	47.0	46.5	47.1	47.6
Age 20–24: %	55.2	54.9	56.1	56.5
Never married: %	20.8	20.5	19.7	19.9
Worked in past 12 months: %	25.2	24.8	25.0	25.3
Years of schooling ^1^	10.604 (2.542)	10.612 (2.518)	10.597 (2.585)	10.620 (2.580)
Bakongo ethnicity: %	35.7	35.1	35.1	35.6
Household wealth: %				
Low	32.7	32.4	30.8	31.3
Medium	33.7	33.8	35.2	35.0
High	33.6	33.8	34.0	33.7
**FTM’s PPFP knowledge and attitudes**				
No. of modern FP methods FTM knows ^1^	6.224 (2.194)			
Approved of FP use in immediate postpartum period: %	73.7			
FP myths rejection index ^1^	19.807 (4.405)			
**FTM’s PPFP perceived norms**				
Injunctive norms index ^1^	10.342 (5.659)			
Descriptive norms: %	11.6			
Normative expectations ^1^	10.972 (2.304)			
Community reaction to PPFP use: %				
Say good things	33.5			
Say bad things	37.7			
Indifferent	28.8			
**FTM’s FP-related behaviors**				
Ever used FP before pregnancy: %	54.2			
Discussed FP with male partner in the immediate postpartum period: %	55.6			
**FTM personal agency**				
PPFP self-efficacy ^1^	18.963 (5.194)			
EBF autonomy: %				60.9
**FTM perceived EBF norms**				
Injunctive norms index ^1^				9.653 (6.153)
Descriptive norms: %				16.2
Normative expectations: %				30.3
**ANC content (a)**				
Adapted index of WHO-recommended actions for positive pregnancy ^1^			7.431 (1.773)	
Index of ANC counseling ^1^			9.228 (2.925)	
Received ANC counseling on EBF: %			92.5	
N	1335	1286	1108	1054

^1^ Mean; (a) Measured at endline; () Standard deviation; na—Not applicable; ANC—Antenatal care; EBF—Exclusive breastfeeding; FP—Family planning; FTM—First-time mother; GEM—Gender-equitable men; IPV—Intimate partner violence; MNH—Maternal and newborn health; PPFP—Postpartum family planning

**Table 2:** Percent of FTMs with specific postpartum family planning outcomes by indicators of the male partner’s gender-equitable attitudes and behaviors, Kinshasa, DRC.

Male Partner Variables	Discussed FP with Partner in Immediate Postpartum Period		Obtained/Bought a FP Method in the Immediate Postpartum Period		Used a Modern FP Method in the Immediate Postpartum Period		Used a Modern Contraceptive 0–11 Months After Childbirth or Pregnancy Loss		
	%		%		%		%		N
GEM Scale									
Low	57.0		26.1		8.7		48.6		675
High	54.1		23.8		8.2		45.8		660
History of IPV perpetration									
Low	56.0		23.8		7.6		45.9		647
High	55.2		26.0		9.3		48.4		688
MH involvement index (a)		***		*				**	
Low	40.6		20.4		8.8		40.3		397
High	61.9		26.8		8.3		50.1		938
Willingness index to perform caregiving activities for infants									
Low	55.5		25.1		8.4		46.0		641
High	55.6		24.8		8.5		48.3		694
Total	55.5		24.9		8.5		47.2		1335

(a) Measured at endline. * *p* < 0.05, ** *p* < 0.01, *** *p* < 0.001

**Table 3:** Mean shared decision-making index and percentage of FTMs with specific maternal and child health outcomes by indicators of the male partner’s gender-equitable attitudes and behaviors, Kinshasa, DRC.

Male Partner Variables	FTM-Reported Shared MNH Decision Making Index		Completion of the MH Continuum of Care		Exclusive Breastfeeding for Six Months (b)	
	Mean (SD)	N	%	N	%	N
GEM Scale	**					
Low	2.808 (2.670)	647	72.8	558	43.1	526
High	3.125 (2.803)	639	74.4	550	37.5	528
History of IPV perpetration						
Low	2.965 (2.675)	620	74.0	538	41.0	505
High	2.967 (2.803)	666	73.2	570	39.7	549
MH involvement index (a)	***		*		***	
Low	2.079 (2.420)	381	69.2	318	28.6	304
High	3.339 (2.783)	905	75.3	790	45.0	750
Willingness index to perform caregiving activities for infants						
Low	2.926 (2.758)	618	74.4	520	41.8	491
High	3.003 (2.726)	669	72.8	588	39.1	563
MNH shared decision-making index						
Low	na		73.4	837	39.6	798
High	na		74.2	271	42.6	256
Total	2.966 (2.741)	1286	73.6	1108	40.3	1054

(a) Measured at endline; (b) Restricted to FTMs whose babies survived for six months or longer; na—Not applicable. Data pertain to FTMs with live births. * *p* < 0.05, ** *p* < 0.01, *** *p* < 0.001

**Table 4:** Results of logistic regression models for selected postpartum family planning outcomes, first-time mothers age 15–24, Kinshasa, DRC.

Independent Variables	Discussed FP with Partner in Immediate Postpartum Period			Obtained/Bought a FP Method in the Immediate Postpartum Period			Used a Modern FP Method in Immediate Postpartum Period			Used a Modern Contraceptive 0–11 Months after Childbirth or Pregnancy Loss		
	AOR		95% CI	AOR		95% CI	AOR		95% CI	AOR		95% CI
**Male partner’s gender-equitable attitudes and behaviors at baseline**												
GEM Scale	1.048		[0.914, 1.203]	0.926		[0.787, 1.089]	0.967		[0.765, 1.222]	1.003		[0.878, 1.147]
History of IPV perpetration	0.968		[0.764, 1.228]	1.159		[0.879, 1.527]	1.256		[0.839, 1.879]	1.114		[0.886, 1.401]
MH involvement index (a)	1.176	***	[1.128, 1.226]	1.015		[0.966, 1.066]	0.986		[0.920, 1.057]	1.039		[0.998, 1.082]
Willingness index to perform caregiving activities for infants	0.962		[0.847, 1.093]	1.006		[0.870, 1.162]	0.841		[0.691, 1.025]	1.047		[0.925, 1.185]
**FTM attitudes and knowledge**												
Approves of PPFP	1.066		[0.755, 1.505]	0.814		[0.539, 1.230]	1.121		[0.611, 2.056]	1.152		[0.824, 1.610]
No. of modern FP methods known	1.067	*	[1.008, 1.129]	1.005		[0.941, 1.074]	0.945		[0.857, 1.042]	1.034		[0.978, 1.092]
No. of FP myths rejected	1.010		[0.981, 1.040]	1.045	*	[1.010, 1.081]	1.055	*	[1.004, 1.108]	1.031	*	[1.003, 1.061]
**FTM perceived norms**												
Injunctive norms index	1.013		[0.987, 1.040]	1.042	*	[1.009, 1.075]	1.028		[0.981, 1.077]	1.026	*	[1.001, 1.053]
Descriptive norms	0.991		[0.681, 1.444]	0.753		[0.480, 1.182]	0.97		[0.493, 1.908]	1.267		[0.879, 1.826]
Normative expectations	1.022		[0.952, 1.097]	0.989		[0.910, 1.074]	0.976		[0.866, 1.099]	1.015		[0.948, 1.087]
Community reaction to PPFP use												
Say bad things	1.068		[0.792, 1.439]	0.952		[0.677, 1.339]	0.756		[0.452, 1.264]	0.881		[0.660, 1.176]
Indifferent	1.119		[0.823, 1.521]	0.452	***	[0.311,0.656]	0.785		[0.475, 1.298]	0.741	*	[0.550,0.997]
**FTM personal agency**												
PPFP Self-efficacy	1.008		[0.980, 1.038]	0.989		[0.955, 1.024]	0.979		[0.932, 1.028]	1.009		[0.981, 1.038]
**Other FTM baseline characteristics**												
Unintended pregnancy	1.187		[0.862, 1.633]	0.988		[0.683, 1.430]	0.986		[0.578, 1.682]	1.004		[0.738, 1.368]
Intervention health zone	2.629	***	[2.053, 3.367]	1.818	***	[1.355, 2.439]	1.970	**	[1.270, 3.054]	1.663	***	[1.303, 2.122]
Age	1.054	*	[1.001, 1.110]	1.046		[0.985, 1.111]	1.035		[0.949, 1.129]	0.960		[0.914, 1.009]
Never married	0.996		[0.738, 1.343]	0.908		[0.635, 1.296]	0.731		[0.424, 1.261]	0.599	***	[0.446, 0.804]
Worked in the past 12 months	0.979		[0.741, 1.292]	0.739		[0.528, 1.033]	1.286		[0.809, 2.044]	1.190		[0.910, 1.557]
FTM’s years of schooling	0.997		[0.947, 1.050]	0.963		[0.907, 1.022]	0.923	*	[0.854, 0.998]	0.954		[0.908, 1.003]
Bakongo ethnicity	1.014		[0.791, 1.299]	0.859		[0.643, 1.148]	1.494		[0.992,2.249]	0.880		[0.692, 1.118]
Household wealth												
Medium	0.887		[0.664, 1.184]	0.660	*	[0.471, 0.926]	1.758	*	[1.045,2.958]	1.103		[0.834, 1.458]
High	0.819		[0.605, 1.108]	0.969		[0.686, 1.368]	2.186	**	[1.274,3.751]	1.074		[0.801, 1.439]
Ever used FP before pregnancy	1.169		[0.914, 1.496]	1.149		[0.859, 1.536]	1.380		[0.896,2.126]	1.364	*	[1.074, 1.733]
**Partner discussion of FP**												
Partner discussion of FP in immediate postpartum period	na			5.462	***	[3.898,7.655]	1.447		[0.931,2.249]	1.535	***	[1.207, 1.951]
Constant	0.049	***	[0.016, 0.154]	0.048	***	[0.013, 0.185]	0.030	***	[0.004, 0.209]	0.188	**	[0.063, 0.568]
Log likelihood	−825.05			−639.17				−364.087				−867.777
Number of FTMs	1335			1335				1335				1335

Data pertain to FTMs who were interviewed at both baseline and endline and whose data could be linked to that of their male partner. (a) Measured at endline. * *p* < 0.05, ** *p* < 0.01, *** *p* < 0.001.

**Table 5:** Results of logistic regression models for selected postpartum family planning outcomes among first-time mothers age 15–24, with interaction terms between the couple’s marital status and the male partner’s gender-equitable attitudes and behaviors, Kinshasa, DRC.

Independent Variables	Discussed FP with Partner in Immediate Postpartum Period			Obtained/Bought a FP Method in the Immediate Postpartum Period			Used a Modern FP Method in Immediate Postpartum Period			Used a Modern Contraceptive 0–11 Months after Childbirth or Pregnancy Loss		
	AOR		95% CI	AOR		95% CI	AOR		95% CI	AOR		95% CI
**Male partner’s gender-equitable attitudes and behavior at baseline**												
GEM Scale	1.102		[0.944, 1.286]	0.852		[0.710, 1.022]	0.871		[0.670, 1.131]	0.967		[0.833, 1.123]
History of IPV perpetration	0.89		[0.682, 1.160]	1.386	*	[1.018, 1.887]	1.263		[0.812, 1.964]	1.001		[0.775, 1.292]
MH involvement index (a)	1.162	***	[1.108, 1.218]	1.001		[0.947, 1.058]	0.959		[0.889, 1.035]	1.037		[0.991, 1.085]
Willingness index to perform caregiving activities for infants	0.886		[0.762, 1.031]	0.943		[0.797, 1.115]	0.778	*	[0.624, 0.970]	0.978		[0.846, 1.130]
**Interaction terms**												
GEM Scale*Never married FTM	0.800		[0.569, 1.124]	1.544	*	[1.022, 2.333]	1.850	*	[1.014, 3.378]	1.229		[0.879, 1.719]
IPV*Never married FTM	1.595		[0.881,2.889]	0.419	*	[0.207, 0.847]	1.129		[0.374, 3.407]	1.828	*	[1.014, 3.295]
MH involvement index*Never married FTM	1.055		[0.957, 1.162]	1.065		[0.951, 1.194]	1.153		[0.970, 1.370]	1.013		[0.924, 1.110]
Willingness*Never married FTM	1.363	*	[1.017, 1.827]	1.269		[0.895, 1.800]	1.567		[0.876,2.801]	1.353	*	[1.005, 1.820]
**FTM baseline characteristics**												
Never married	0.563		[0.262, 1.212]	0.996		[0.388,2.558]	0.276		[0.060, 1.278]	0.405	*	[0.192, 0.858]
Constant	0.059	***	[0.019, 0.190]	0.045	***	[0.011, 0.175]	0.035	***	[0.005, 0.251]	0.203	**	[0.066, 0.621]
Log likelihood	−820.176			−631.734			−359.457			−863.448		
Number of FTMs	1335			1335			1335			1335		

Data pertain to FTMs who were interviewed at both baseline and endline and whose data could be linked to that of their male partner. Regressions control for all other variables shown in Table 4. (a) Measured at endline. * *p* < 0.05, ** *p* < 0.01, *** *p* < 0.001.

**Table 6:** Results of multivariable linear regression models of the index of shared decision making about maternal and newborn health issues, first-time mothers age 15–24, Kinshasa, DRC.

Independent Variables	Model 1			Model 2			Model 3		
	Adj. Coef.		95% CI	Adj. Coef.		95% CI	Adj. Coef.		95% CI
**Male partner’s gender-equitable attitudes and behavior at baseline**									
GEM Scale	0.134		[−0.035, 0.303]	0.093		[−0.076, 0.262]	0.073		[−0.117, 0.262]
History of IPV perpetration	0.110		[−0.181, 0.401]	0.170		[−0.120, 0.461]	0.124		[−0.202, 0.450]
MNH involvement index (a)	0.244	***	[0.196, 0.292]	0.222	***	[0.173, 0.272]	0.229	***	[0.173, 0.286]
Willingness index to perform routine caregiving activities for infants	−0.059		[−0.217, 0.100]	−0.096		[−0.254, 0.063]	−0.131		[−0.314, 0.053]
**Interaction terms**									
GEM Scale*Never married FTM							0.108		[−0.311, 0.527]
IPV*Never married FTM							0.243		[−0.483, 0.969]
MNH involvement index*Never married FTM							−0.027		[−0.141, 0.087]
Willingness*Never married FTM							0.148		[−0.217, 0.513]
**Other FTM baseline characteristics**									
Unintended pregnancy				−0.573	**	[−0.958,−0.187]	−0.567	**	[−0.954,−0.179]
Intervention health zone				0.079		[−0.215, 0.374]	0.081		[−0.215, 0.376]
Age 20–24				0.491	**	[0.178, 0.805]	0.500	**	[0.185, 0.814]
Never married				−0.335		[−0.703, 0.034]	−0.284		[−1.200, 0.632]
Worked in the past 12 months				−0.016		[−0.353, 0.322]	−0.010		[−0.349, 0.328]
FTM’s years of schooling				−0.015		[−0.077, 0.046]	−0.016		[−0.078, 0.045]
Bakongo ethnicity				−0.225		[−0.531, 0.081]	−0.235		[−0.542, 0.072]
Household wealth									
Medium				−0.207		[−0.564, 0.150]	−0.203		[−0.561, 0.155]
High				−0.225		[−0.596, 0.147]	−0.231		[−0.603, 0.142]
Constant	1.214	***	[0.813, 1.615]	1.950	**	[1.130,2.770]	1.106	**	[1.077,2.785]
Log likelihood	−3071.452			−3055.339			−3054.612		
Number of FTMs	1286			1286			1286		

Data pertain to FTMs with a live birth who were interviewed at both baseline and endline, whose data could be linked to that of their male partner. (a) Measured at endline. Data pertain to FTM reports of shared decision making. ** *p* < 0.01, *** *p* < 0.001.

**Table 7:** Results of multivariable logistic regression models of completion of the maternal health continuum of care, first-time mothers age 15–24, Kinshasa, DRC.

Independent Variables	Model 1			Model 2			Model 3			Model 4		
	AOR		95% CI	AOR		95% CI	AOR		95% CI	AOR		95% CI
**Male partner’s gender-equitable attitudes and behavior at baseline**												
GEM Scale	1.129		[0.961, 1.325]	1.041		[0.869, 1.247]	1.118		[0.912, 1.370]	1.126		[0.918, 1.381]
History of IPV perpetration	0.981		[0.748, 1.286]	0.876		[0.648, 1.185]	0.900		[0.643, 1.259]	0.895		[0.640, 1.253]
MNH involvement index (a)	1.024		[0.978, 1.072]	1.022		[0.968, 1.079]	1.004		[0.945, 1.067]	1.005		[0.946, 1.068]
Willingness index to perform caregiving activities for infants	0.990		[0.853, 1.149]	0.893		[0.752, 1.061]	0.832		[0.679, 1.020]	0.834		[0.680, 1.022]
No. of shared MNH decisions	0.962		[0.910, 1.016]	0.970		[0.911, 1.032]	0.971		[0.911, 1.034]	0.948		[0.887, 1.012]
**Interaction terms**												
GEM Scale*Never married FTM							0.725		[0.468, 1.124]	0.676		[0.431, 1.060]
IPV*Never married FTM							0.906		[0.414, 1.982]	0.815		[0.369, 1.797]
MNH involvement index*Never married FTM							1.092		[0.965, 1.237]	1.088		[0.960, 1.234]
Willingness*Never married FTM							1.287		[0.875, 1.893]	1.234		[0.835, 1.824]
Shared MNH decisions*Never married FTM										1.370		[0.996, 1.883]
**Other FTM baseline characteristics**												
Unintended pregnancy				1.050		[0.703, 1.570]	1.021		[0.682, 1.528]	0.994		[0.663, 1.490]
Intervention health zone				0.620	**	[0.455, 0.843]	0.617	**	[0.452, 0.842]	0.618	**	[0.453, 0.844]
Age 20–24				1.202		[0.871, 1.658]	1.197		[0.866, 1.655]	1.212		[0.876, 1.678]
Never married				1.006		[0.681, 1.486]	0.623		[0.232, 1.672]	0.559		[0.206, 1.514]
Worked in the past 12 months				1.042		[0.733, 1.481]	1.035		[0.727, 1.475]	1.050		[0.736, 1.498]
FTM’s years of schooling				1.040		[0.980, 1.104]	1.039		[0.979, 1.104]	1.040		[0.979, 1.104]
Bakongo ethnicity				0.790		[0.579, 1.077]	0.796		[0.583, 1.086]	0.811		[0.594, 1.108]
Household wealth												
Medium				1.015		[0.708, 1.455]	0.989		[0.688, 1.421]	0.989		[0.687, 1.422]
High				1.086		[0.736, 1.602]	1.056		[0.714, 1.563]	1.044		[0.705, 1.546]
**ANC content**												
Index of WHO-recommended actions for positive pregnancy (a)				1.547	***	[1.317, 1.816]	1.567	***	[1.332, 1.845]	1.553	***	[1.321, 1.826]
Index of ANC counseling (a)				1.126	***	[1.050, 1.208]	1.125	***	[1.049, 1.207]	1.131	***	[1.054, 1.214]
Constant	2.555	***	[1.742,3.746]	0.028	***	[0.007, 0.102]	0.030	***	[0.008, 0.114]	0.032	***	[0.008, 0.122]
Log likelihood	−637.797			−545.942			−542.869			540.144		
Number of FTMs	1108			1108			1108			1108		

Data pertain to FTMs who were interviewed at both baseline and endline and whose data could be linked to that of their male partner. (a) Measured at endline. ** *p* < 0.01, *** *p* < 0.001.

**Table 8:** Results of multivariable logistic regression models of exclusive breastfeeding for six months among first-time mothers age 15–24, Kinshasa, DRC.

Independent Variables	Model 1			Model 2			Model 3			Model 4		
	AOR		95% CI	AOR		95% CI	AOR		95% CI	AOR		95% CI
**Male partner’s gender-equitable attitudes and behavior at baseline**												
GEM Scale	0.887		[0.763, 1.031]	0.891		[0.761, 1.043]	0.969		[0.812, 1.157]	0.968		[0.811, 1.155]
History of IPV perpetration	0.972		[0.754, 1.252]	0.946		[0.727, 1.232]	0.952		[0.708, 1.280]	0.952		[0.708, 1.281]
MNH involvement index (a)	1.110	***	[1.062, 1.161]	1.107	***	[1.054, 1.162]	1.119	***	[1.058, 1.184]	1.119	***	[1.058, 1.183]
Willingness index to perform caregiving activities for infants	0.864	*	[0.751, 0.995]	0.868		[0.749, 1.005]	0.806	*	[0.677, 0.959]	0.805	*	[0.677, 0.959]
No. of shared MNH decisions	1.079	**	[1.024, 1.138]	1.080	**	[1.021, 1.142]	1.079	**	[1.020, 1.141]	1.085	**	[1.022, 1.151]
**Interaction terms**												
GEM Scale*Never married FTM							0.676		[0.457, 1.001]	0.688		[0.463, 1.023]
IPV*Never married FTM							0.962		[0.491, 1.885]	0.982		[0.499, 1.934]
MNH involvement index*Never married FTM							0.963		[0.862, 1.077]	0.965		[0.863, 1.079]
Willingness*Never married FTM							1.275		[0.920, 1.769]	1.288		[0.927, 1.791]
Shared MNH decisions*Never married FTM										0.956		[0.799, 1.143]
**FTM perceived EBF norms at baseline**												
Injunctive norms index				1.017		[0.993, 1.042]	1.018		[0.994, 1.043]	1.018		[0.994, 1.043]
Descriptive norms				1.359		[0.937, 1.971]	1.345		[0.926, 1.953]	1.344		[0.926, 1.952]
Normative expectations				1.037		[0.757, 1.421]	1.039		[0.757, 1.427]	1.041		[0.758, 1.430]
**FTM’s personal agency at baseline**												
EBF autonomy				2.389	***	[1.808,3.157]	2.466	***	[1.862,3.268]	2.471	***	[1.865,3.274]
**Other FTM baseline characteristics**												
Unintended pregnancy				1.155		[0.813, 1.641]	1.156		[0.812, 1.647]	1.163		[0.816, 1.658]
Intervention health zone				1.296		[0.988, 1.701]	1.292		[0.984, 1.698]	1.292		[0.984, 1.697]
Age 20–24				1.154		[0.866, 1.538]	1.147		[0.858, 1.532]	1.145		[0.857, 1.530]
Never married				1.238		[0.879, 1.745]	1.607		[0.652,3.961]	1.631		[0.660,4.028]
Worked in the past 12 months				0.961		[0.709, 1.302]	0.947		[0.697, 1.286]	0.945		[0.696, 1.283]
FTM’s years of schooling				0.979		[0.927, 1.034]	0.978		[0.926, 1.034]	0.978		[0.926, 1.034]
Bakongo ethnicity				1.280		[0.971, 1.687]	1.284		[0.973, 1.695]	1.278		[0.968, 1.688]
Household wealth												
Medium				1.013		[0.732, 1.403]	0.993		[0.716, 1.377]	0.993		[0.716, 1.377]
High				1.160		[0.824, 1.633]	1.140		[0.808, 1.608]	1.141		[0.809, 1.609]
Received ANC counseling on EBF				2.328	**	[1.269,4.268]	2.352	**	[1.279,4.326]	2.336	**	[1.270,4.297]
Constant	0.289	***	[0.198, 0.422]	0.047	***	[0.018, 0.121]	0.043	***	[0.016, 0.115]	0.043	***	[0.016, 0.114]
Log likelihood	691.856			−654.996			−651.653			651.53		
Number of FTMs	1054			1054			1054			1054		

Data pertain to FTMs who were interviewed at both baseline and endline, whose data could be linked to that of their male partner, and whose baby survived for 6 or more months. (a) Measured at endline. * *p* < 0.05, ** *p* < 0.01, *** *p* < 0.001.

## Data Availability

The datasets used and/or analyzed during the current study are available from the corresponding author on reasonable request.

## Supplementary Materials

The following supporting information can be downloaded at: https://www.mdpi.com/article/10.3390/ijerph191912182/s1, Table S1: Percent distribution of first-time mothers aged 15–24 who were completely interviewed at baseline and mean indices of interest, by loss-to-follow-up, Kinshasa, DRC; Table S2: Percent distribution of first-time mothers age 15–24 who had live births by their reported pattern of decision making about specific maternal and newborn health issues, Kinshasa, DRC; Table S3: Percent distribution of first-time mothers age 15–24 by their male partner’s agreement with items comprising the Gender-equitable Men (GEM) scale, Kinshasa, DRC; Table S4: Percent distribution of first-time mothers aged 15–24 by components of the index of male partner involvement in maternal health, Kinshasa, DRC; Table S5: Percent distribution of first-time mothers age 15–24 by their male partner’s willingness to engage in specific routine childcare activities for their baby, Kinshasa, DRC; Table S6: Percentage of first-time mothers age 15–24 who experienced specific components of the adapted index of World Health Organization-recommended actions for a positive pregnancy experience during antenatal care, Kinshasa, DRC; Table S7: Percentage of first-time mothers age 15–24 who received antenatal care counselling on specific topics, Kinshasa, DRC; Table S8: Percent distribution of first-time mothers age 15–24 by the male partner’s reported decision making pattern and the first-time mothers’ reported joint decision making about specific maternal and newborn health issues, Kinshasa, DRC; Table S9: Results of multivariable linear regression models of the index of shared decision making about maternal and newborn health issues derived from male partners’ reports, first-time mothers age 15–24, Kinshasa, DRC.
