# Supplementary material for: Association of Male Partners’ Gender-Equitable Attitudes and Behaviors with Young Mothers’ Postpartum Family Planning and Maternal Health Outcomes in Kinshasa, DRC
Source: Int J Environ Res Public Health. 2022 Sep 26;19(19):12182. doi: 10.3390/ijerph191912182 (PMC9565980; doi:10.3390/ijerph191912182)
Supplement: Supplementary file 1 [file ijerph-19-12182-s001.zip › ijerph-1903917-supplementary.pdf]

International Journal of
Environmental Research and Public Health

MDPI

Supplementary Materials

Table S1: Percent distribution of first-time mothers age 15-24 who were completely interviewed at baseline and mean indices of interest, by loss-to-follow-up, Kinshasa, DRC

Background Characteristics	FTM LTFU	FTM Interviewed at Edline and Male Partner LTFU	FTM Interviewed at Endline and Male Partner Not Named at Baseline	Both FTM and Male Partner Retained	Total	P-value
			Percent Distribution			
**Unintended pregnancy**						<0.001
Intended	22.6	17.5	8.0	19.6	18.6	
Unintended	77.4	82.5	92.0	80.4	81.4	
**Health zone**						<0.001
Control	48.2	39.4	48.7	53.0	49.9	
Intervention	51.8	60.6	51.3	47.0	50.1	
**Age group**						<0.001
15-19	60.9	56.8	73.3	55.1	58.7	
20-24	39.1	43.2	26.7	44.9	41.3	
**Marital status**						<0.001
Ever married/formally engaged	74.4	61.3	35.0	79.3	70.6	
Never married	25.6	38.7	65.0	20.7	29.4	
**Worked in the past 12 months**						0.306
No	65.1	60.3	67.3	63.4	63.9	
Yes	34.9	39.7	32.7	36.6	36.1	
**Bakongo ethnicity**						0.003
No	75.0	78.4	65.7	71.7	72.4	
Yes	25.0	21.6	34.3	28.3	27.6	
**Household wealth**						0.013
Low	41.2	34.9	36.7	32.7	35.2	
Medium	32.6	36.3	33.0	33.6	33.6	
High	26.2	28.7	30.3	33.7	31.2	
**Approves of PPFP use**						0.880
No	28.0	27.1	26.3	26.1	26.7	
Yes	72.0	72.9	73.7	73.9	73.3	
**Descriptive norms about PPFP use**						0.262
No	90.3	88.0	85.7	88.5	88.5	
Yes	9.7	12.0	14.3	11.5	11.5	
**Perceived community reaction to PPFP use**						0.232
Bad/indifferent	70.6	71.2	67.3	66.6	68.1	
Good	29.4	28.8	32.7	33.4	31.9	
**Ever used FP**						0.004
Never used	50.8	47.9	58.3	45.9	48.7	
Traditional methods	15.7	15.1	14.0	14.9	15.0	
Modern methods	33.5	37.0	27.7	39.2	36.3	
**Injunctive norms about EBF**						0.163
Fewer referents	38.5	33.2	33.3	38.4	37.2	
Most referents	61.5	66.8	66.7	61.6	62.8	
**Descriptive norms about EBF**						0.392
About half or fewer	92.7	90.4	93.0	90.8	91.4	
All/more than half	7.3	9.6	7.0	9.2	8.6	

**Background Characteristics**	**FTM LTFU**	**FTM Interviewed at Edline and Male Partner LTFU**	**FTM Interviewed at Endline and Male Partner Not Named at Baseline**	**Both FTM and Male Partner Retained**	**Total**	**P-value**
**Normative expectations about EBF**						0.891
Fewer referents	70.4	68.8	68.0	68.9	69.1	
Most referents	29.6	31.2	32.0	31.1	30.9	
Total	100.0	100.0	100.0	100.0	100.0	
			**Mean Indices (SD)**			
No. of years of schooling	10.1 (2.610)	10.3 (2.567)	9.3 (3.020)	10.6 (2.572)	10.3 (2.668)	<0.001
No. of modern FP methods known	7.5 (2.576)	7.3 (2.602)	7.3 (2.716)	7.9 (2.459)	7.7 (2.545)	<0.001
FP myths rejection index	19.6 (4.330)	19.7 (4.277)	20.0 (4.440)	19.8 (4.419)	19.8 (4.385)	0.702
PPFP self-efficacy index	18.7 (5.536)	19.1 (4.928)	18.8 (5.321)	19.0 (5.178)	18.9 (5.241)	0.738
EBF injunctive norms index	9.8 (6.120)	10.4 (6.164)	9.9 (6.150)	9.6 (6.171)	9.8 (6.158)	0.301
**N**	**504**	**292**	**300**	**1335**	**2431**	

Source: Momentum 2018 Baseline Survey and 2020 Endline Survey

EBF Exclusive breastfeeding
FP Family planning
FTM First-time mother
LTFU Lost to follow-up
PPFP Postpartum family planning
SD Standard deviation

Table S2: Percent distribution of first-time mothers age 15-24 who had live births by their reported pattern of decision making about specific maternal and newborn health issues, Kinshasa, DRC

Maternal and Newborn Health Decision	Percent	Number
**When to start seeking antenatal care**		
Respondent	23.5	314
Husband/partner	27.9	373
Respondent and husband/partner jointly	39.7	530
Someone else	8.8	118
**Number of antenatal visits**		
Respondent	30.6	393
Husband/partner	12.1	156
Respondent and husband/partner jointly	29.2	376
Someone else	28.1	361
**Where to deliver the baby**		
Respondent	33.6	432
Husband/partner	23.7	305
Respondent and husband/partner jointly	27.1	349
Someone else	15.6	200
**How soon to start breastfeeding the newborn**		
Respondent	60.6	779
Husband/partner	4.0	52
Respondent and husband/partner jointly	19.9	256
Someone else	15.5	199
**Whether to practice exclusive breastfeeding**		
Respondent	50.7	652
Husband/partner	6.6	85
Respondent and husband/partner jointly	24.3	312
Someone else	18.4	237
**Umbilical cord care**		
Respondent	21.8	280
Husband/partner	2.6	33
Respondent and husband/partner jointly	21.7	279
Someone else	54.0	694
**When to seek care and treatment for danger signs of the mother**		
Respondent	29.9	384
Husband/partner	16.4	211
Respondent and husband/partner jointly	42.9	552
Someone else	10.8	139
**How long to wait after childbirth before attempting another pregnancy**		
Respondent	48.4	623
Husband/partner	5.6	72
Respondent and husband/partner jointly	43.5	559
Someone else	2.5	32
**When to seek care and treatment for danger signs of the newborn**		
Respondent	27.8	358
Husband/partner	14.2	183
Respondent and husband/partner jointly	48.5	624
Someone else	9.4	121
**Total**	**100.0**	**1286**

Source: Momentum 2020 Endline Survey
Column totals may not add up to 100 due to rounding.

Table S3: Percent distribution of first-time mothers age 15-24 by their male partner's agreement with items comprising the Gender-equitable Men (GEM) scale, Kinshasa, DRC

GEM Scale Items	Percent	Number
**A woman's most important role is to take care of her home and cook for her family.**		
Totally agree	63.1	843
Partially agree	10.5	140
Disagree	26.4	352
**Men need sex more than women do.**		
Totally agree	46.9	626
Partially agree	19.0	254
Disagree	34.1	455
**You don't talk about sex; you just do it.**		
Totally agree	21.6	288
Partially agree	12.8	171
Disagree	65.6	876
**There are times when a woman deserves to be beaten.**		
Totally agree	26.8	358
Partially agree	18.1	241
Disagree	55.1	736
**Changing diapers, giving a bath, and feeding kids is the mother's responsibility.**		
Totally agree	61.9	826
Partially agree	14.2	190
Disagree	23.9	319
**It is a woman's responsibility to avoid getting pregnant.**		
Totally agree	35.8	478
Partially agree	19.4	259
Disagree	44.8	598
**A man should have the final word about decisions in his home.**		
Totally agree	76.7	1024
Partially agree	13.1	175
Disagree	10.2	136
**Men are always ready to have sex.**		
Totally agree	47.0	628
Partially agree	20.5	274
Disagree	32.4	433
**A woman should tolerate violence to keep her family together.**		
Totally agree	37.5	500
Partially agree	18.5	247
Disagree	44.0	588
**If someone insults me, I will defend my reputation, with force if I have to.**		
Totally agree	20.5	274
Partially agree	16.1	215
Disagree	63.4	846
**To be a man, you need to be tough.**		
Totally agree	44.6	595
Partially agree	16.9	225
Disagree	38.6	515
**Total**	**100.0**	**1335**

Source: Momentum 2018 Baseline Survey
Column percentages may not add up to 100 due to rounding.

Table S4: Percent distribution of first-time mothers age 15-24 by components of the index of male partner involvement in maternal health, Kinshasa, DRC

Components of Male Partner Involvement	Percent	Number
**Present at pregnancy test/confirmation**		
No	78.7	1051
Yes	21.3	284
**Present at one or more ANC visits**		
No	85.1	1136
Yes	14.9	199
**Attend one or more group education classes about pregnancy and fatherhood**		
No	80.6	1076
Yes	19.4	259
**Present at childbirth/delivery or when FTM lost the pregnancy**		
No	75.5	1008
Yes	24.5	327
**Found out information about the pregnancy**		
No	57.8	771
Yes	42.2	564
**Decision making about ANC**		
No	41.6	555
Yes	58.4	780
**Making a birth plan**		
No	38.7	517
Yes	61.3	818
**Saving money for emergencies**		
No	29.4	392
Yes	70.6	943
**Arranging transport for delivery**		
No	46.5	621
Yes	53.5	714
**Deciding on skilled attendance at delivery**		
No	69.8	932
Yes	30.2	403
**Encouraging exclusive breastfeeding**		
No	51.5	687
Yes	48.5	648
**Other**		
No	86.0	1148
Yes	14.0	187
**Total**	**100.0**	**1335**

Source: Momentum 2020 Endline Survey
Column percentages may not add up to 100 due to rounding.
ANC Antenatal care

Table S5: Percent distribution of first-time mothers age 15-24 by their male partner's willingness to engage in specific routine childcare activities for their baby, Kinshasa, DRC

Routine Child Care Activities	Percent	Number
**Changing the baby's diapers**		
Not at all	9.4	126
Somewhat unwilling	4.3	58
Undecided	3.2	43
Somewhat willing	34.8	465
Extremely willing	48.2	643
**Helping/supporting feeding**		
Not at all	2.5	34
Somewhat unwilling	1.7	23
Undecided	1.1	15
Somewhat willing	32.1	428
Extremely willing	62.5	835
**Helping when baby cries**		
Not at all	1.9	25
Somewhat unwilling	0.3	4
Undecided	1.1	15
Somewhat willing	35.4	472
Extremely willing	61.3	819
**Bathing the baby**		
Not at all	9.7	129
Somewhat unwilling	4.1	55
Undecided	3.4	45
Somewhat willing	37.3	498
Extremely willing	45.5	608
**Looking after the baby when the mother goes out or is at work**		
Not at all	2.6	35
Somewhat unwilling	1.0	13
Undecided	2.2	30
Somewhat willing	33.9	453
Extremely willing	60.2	804
**Washing the baby's clothes**		
Not at all	16.6	222
Somewhat unwilling	3.9	52
Undecided	4.6	62
Somewhat willing	36.6	489
Extremely willing	38.2	510
**Cooking or preparing food**		
Not at all	18.0	240
Somewhat unwilling	3.7	50
Undecided	4.2	56
Somewhat willing	37.3	498
Extremely willing	36.8	491
**House cleaning**		
Not at all	16.8	224
Somewhat unwilling	4.4	59
Undecided	4.1	55
Somewhat willing	41.3	552
Extremely willing	33.3	445
**Putting the baby to sleep/bed**		
Not at all	2.2	30
Somewhat unwilling	1.3	17

Routine Child Care Activities	Percent	Number
Undecided	1.1	15
Somewhat willing	36.7	490
Extremely willing	58.7	783
**Staying home when the child is sick**		
Not at all	7.6	101
Somewhat unwilling	2.5	33
Undecided	2.6	35
Somewhat willing	34.4	459
Extremely willing	53.0	707
**Taking the baby to the doctor**		
Not at all	0.7	9
Somewhat unwilling	0.4	5
Undecided	0.5	7
Somewhat willing	25.7	343
Extremely willing	72.7	971
**Total**	**100.0**	**1335**

Source: Momentum 2018 Baseline Survey
Column percentages may not add up to 100 due to rounding.

Table S6: Percentage of first-time mothers age 15-24 who experienced specific components of the adapted index of World Health Organization-recommended actions for a positive pregnancy experience during antenatal care, Kinshasa, DRC

ANC Actions by Provider	Percent	Number
**Weigh you**		
No	5.8	77
Yes	94.2	1258
**Feel your abdomen**		
No	6.3	84
Yes	93.7	1251
**Measure blood pressure**		
No	6.4	86
Yes	93.6	1249
**Take a urine sample**		
No	8.4	112
Yes	91.6	1223
**Take a blood sample**		
No	9.3	124
Yes	90.7	1211
**Give you/ask you to buy iron tablets/syrup**		
No	6.9	92
Yes	93.1	1243
**Give you/ask you to buy SP/Fansidar**		
No	7.3	97
Yes	92.7	1238
**Listened to the baby's heartbeat**		
No	8.5	114
Yes	91.5	1221
**Total**	**100.0**	**1335**

Source: Momentum 2020 Endline Survey
Column percentages may not add up to 100 due to rounding.
SP Sulphadoxine Pyrimethamine

Table S7: Percentage of first-time mothers age 15-24 who received antenatal care counselling on specific topics, Kinshasa, DRC

Antenatal Care Counseling Topics	Percent	Number
**Exclusive breastfeeding**		
No	12.0	133
Yes	88.0	975
**Newborn care**		
No	14.7	163
Yes	85.3	945
**Sleeping under an insecticide-treated net**		
No	6.2	69
Yes	93.8	1039
**Birth preparedness**		
No	13.5	150
Yes	86.5	958
**Delivery with skilled birth attendant**		
No	19.2	213
Yes	80.8	895
**Birth spacing**		
No	19.9	221
Yes	80.1	887
**Family planning**		
No	26.3	291
Yes	73.7	817
**Prevention of mother-to-child transmission of HIV**		
No	16.4	182
Yes	83.6	926
**Foods the FTM should eat**		
No	13.0	144
Yes	87.0	964
**Obstetric danger signs**		
No	15.8	175
Yes	84.2	933
**Newborn danger signs**		
No	20.0	222
Yes	80.0	886
**Total**	**100.0**	**1108**

Source: Momentum 2020 Endline Survey
Column percentages may not add up to 100 due to rounding.

Table S8: Percent distribution of first-time mothers age 15-24 by the male partner's reported decision making pattern and the first-time mothers' reported joint decision making about specific maternal and newborn health issues, Kinshasa, DRC

	Male Partner-reported Decision-making Pattern						
**FTM-reported Joint Decision Making**	**Male Partner Alone**	**FTM Alone**	**Male Partner and FTM Jointly**	**Some-one Else**	**Other**	**Total**	N
**When to start seeking ANC ***							
No	49.7	21.8	18.3	7.3	3.0	100.0	662
Yes	43.9	19.7	25.6	8.7	2.0	100.0	446
**Number of ANC visits ****							
No	25.6	18.3	15.3	25.4	15.4	100.0	777
Yes	17.2	24.2	18.4	27.5	12.7	100.0	331
**Where to deliver the baby**							
No	39.4	31.4	15.0	9.2	5.0	100.0	802
Yes	31.7	37.9	17.6	7.8	4.9	100.0	306
**How soon to start breastfeeding**							
No	13.8	49.5	16.5	14.7	5.6	100.0	887
Yes	13.1	57.5	15.4	9.5	4.5	100.0	221
**Whether to practice EBF**							
No	16.4	35.9	20.6	20.1	7.1	100.0	831
Yes	15.5	39.4	17.0	19.9	8.3	100.0	277
**Umbilical cord care *****							
No	11.4	16.4	11.8	39.1	21.3	100.0	865
Yes	9.1	35.4	13.2	26.7	15.6	100.0	243
**Care seeking for obstetric danger signs *****							
No	55.7	7.3	25.5	9.1	2.4	100.0	628
Yes	47.1	13.1	31.3	6.5	2.1	100.0	480
**Birth spacing *****							
No	61.7	3.4	34.1	0.5	0.3	100.0	619
Yes	41.1	4.3	54.0	0.4	0.2	100.0	489
**Care seeking for newborn danger signs**							
No	55.9	12.4	22.7	6.8	2.2	100.0	555
Yes	52.4	11.6	26.0	8.0	2.0	100.0	553
N							1108

Source: Momentum 2020 Endline Survey
Row percentages may not add up to 100 due to rounding. Data pertain to FTMs with live births and couples with no missing data on variables examined in this study.
* $p < 0.05$, ** $p < 0.01$, *** $p < 0.001$

Table S9: Results of multivariable linear regression models of the index of shared decision making about maternal and newborn health issues derived from male partners' reports, first-time mothers age 15-24, Kinshasa, DRC

	Model 1			Model 2			Model 3		
**Independent Variables**	**Adj. Coef.**		**95% CI**	**Adj. Coef.**		**95% CI**	**Adj. Coef.**		**95% CI**
**Male partner's gender-equitable attitudes and behavior at baseline**									
GEM Scale	0.346	***	[0.180,0.512]	0.286	***	[0.121,0.451]	0.289	**	[0.105,0.474]
History of IPV perpetration	-0.149		[-0.433,0.136]	-0.067		[-0.348,0.213]	-0.190		[-0.502,0.123]
MH involvement index (a)	0.101	***	[0.053,0.149]	0.059	*	[0.010,0.108]	0.072	*	[0.016,0.128]
Willingness index to perform routine caregiving activities for infants	0.195	*	[0.039,0.351]	0.119		[-0.036,0.274]	0.090		[-0.089,0.270]
**Interaction terms**									
GEM Scale*Never married FTM							-0.009		[-0.417,0.400]
IPV*Never married FTM							0.644		[-0.068,1.356]
MH involvement index*Never married FTM							-0.052		[-0.166,0.062]
Willingness*Never married FTM							0.132		[-0.223,0.488]
**Other FTM baseline characteristics**									
Unintended pregnancy				-0.543	**	[-0.910, -0.177]	-0.524	**	[-0.892, -0.156]
Intervention health zone				0.051		[-0.232,0.335]	0.052		[-0.232,0.336]
Age 20-24				0.234		[-0.069,0.538]	0.251		[-0.053,0.555]
Never married				-0.927	***	[-1.287, -0.568]	-0.939	*	[-1.862, -0.017]
Worked in the past 12 months				0.111		[-0.213,0.436]	0.100		[-0.225,0.426]
FTM's years of schooling				0.049		[-0.009,0.106]	0.046		[-0.012,0.103]
Bakongo ethnicity				0.147		[-0.148,0.441]	0.133		[-0.162,0.428]
Household wealth									
Medium				0.020		-0.325,0.366]	0.031		[-0.315,0.377]
High				0.064		[-0.298,0.425]	0.055		[-0.308,0.417]
Constant	1.026	***	[0.624,1.428]	1.124	**	[0.345,1.904]	1.106	**	[0.297,1.916]
Log likelihood	-2533.69			-2505.72			-2503.39		
Number of FTMs	1108			1108			1108		

Data pertain to FTMs who were interviewed at both baseline and endline and whose data could be linked to that of their male partner.
(a) Measured at endline; na - Not applicable; GEM - Gender-equitable men; IPV - Intimate partner violence; MH - Maternal health
* $p < 0.05$, ** $p < 0.01$, *** $p < 0.001$
